# Differential Dynamics of the Ruminal Microbiome of Jersey Cows in a Heat Stress Environment

**DOI:** 10.3390/ani10071127

**Published:** 2020-07-02

**Authors:** Dong-Hyeon Kim, Myung-Hoo Kim, Sang-Bum Kim, Jun-Kyu Son, Ji-Hwan Lee, Sang-Seok Joo, Bon-Hee Gu, Tansol Park, Beom-Young Park, Eun-Tae Kim

**Affiliations:** 1 Dairy Science Division, National Institute of Animal Science, Rural Development Administration, Cheonan 31000, Korea; kimdh3465@korea.kr (D.-H.K.); junkyuson@korea.kr (J.-K.S.); leejh6735@korea.kr (J.-H.L.); byp5252@korea.kr (B.-Y.P.); 2 Department of Animal Science, College of Natural Resources & Life Science, Pusan National University, Miryang 50463, Korea; mhkim18@pusan.ac.kr (M.-H.K.); ssjoo7680@gmail.com (S.-S.J.); 3 Rural Development Administration, Jeonju 54875, Korea; sangbkim@korea.kr; 4 Life and Industry Convergence Institute, College of Natural Resources & Life Science, Miryang 50463, Korea; g.bonhee@gmail.com; 5 Department of Animal Science, Ohio states University, Columbus, OH 43210, USA; park.1777@osu.edu

**Keywords:** ruminal microbiome, heat stress, Jersey, Holstein, KEGG pathways

## Abstract

**Simple Summary:**

Recently, it has become apparent that the microbiome is essential to health and affects practically every aspect of physiology. The rumen contains highly dense and diverse microbial communities, which can impact health through their composition, diversity, and assembly. Nevertheless, the diversity and function of rumen microbes have not been fully described. Therefore, this study aims to identify differences in the functional attributes and metabolites of rumen microbiota to heat stress by metagenomics and metabolomics analyses. We observed differences in biological changes, as well as changes in rumen metabolites and metabolic pathways depending on the breed of cow. In addition, significant changes in rumen bacterial taxa and functional gene abundance were observed. Overall, the findings of this study improve our understanding of heat-vulnerable ruminal bacteria and related genes.

**Abstract:**

The microbial community within the rumen can be changed and shaped by heat stress. Accumulating data have suggested that different breeds of dairy cows have differential heat stress resistance; however, the underlying mechanism by which nonanimal factors contribute to heat stress are yet to be understood. This study is designed to determine changes in the rumen microbiome of Holstein and Jersey cows to normal and heat stress conditions. Under heat stress conditions, Holstein cows had a significantly higher respiration rate than Jersey cows. Heat stress increased the rectal temperature of Holstein but not Jersey cows. In the Kyoto encyclopedia of genes and genomes (KEGG) pathway analysis, Jersey cows had a significantly higher proportion of genes associated with energy metabolism in the normal condition than that with other treatments. Linear discriminant analysis effect size (LEfSe) results identified six taxa as distinguishing taxa between normal and heat stress conditions in Holstein cows; in Jersey cows, 29 such taxa were identified. Changes in the rumen bacterial taxa were more sensitive to heat stress in Jersey cows than in Holstein cows, suggesting that the rumen mechanism is different in both breeds in adapting to heat stress. Collectively, distinct changes in rumen bacterial taxa and functional gene abundance in Jersey cows may be associated with better adaptation ability to heat stress.

## 1. Introduction

Recently, with the continual increase in global greenhouse gas emissions, climate change has become a major global issue for agricultural land and livestock animal production. According to the IPCC (International Panel on Climate Change) climate assessment report, environmental temperatures are projected to rise anywhere between 1.4–5.8 °C over the period from 1990 to 2100 [1]. Recently, the climate in West Asia has been changing from a humid subtropical climate to a tropical climate [2]. This climate change will result in a reduction in the productivity of livestock animals; thus, the development of strategies for rearing livestock animals in ambient temperature is needed to overcome the negative impact of climate change.

Numerous physiological changes occur in the digestive system, acid-base chemical reactions, and blood hormones during hot and humid weather conditions [3]. Temperature-sensitive neurons are located throughout the animal body; these neurons send information to the hypothalamus, which invokes numerous physiological, anatomical, and behavioral changes in an attempt to maintain heat balance [4,5,6]. Various heat stress responses cause changes in a cow’s physiology and productivity, including reduced feed intake [7], lower milk yield [8] and milk quality [9], and compromised immune response [10,11]. Consequently, changes in the ambient temperature lead to increased management costs and reduced profits for the animal industry [12].

In the Republic of Korea, the Holstein breed makes up approximately 99% of the dairy population and is the most important livestock species in Korea’s dairy industry [13]. In general, Holstein cows produce a relatively high amount of milk; however, they are sensitive to environmental changes, such as ambient temperature, humidity, airflow condition, and amount of solar radiation [14]. Interestingly, the susceptibility of cows to heat stress results in a reduced amount of milk production and feed intake. Several researchers have reported that Jersey cows, which are genetically different from Holstein cows [15], have a distinct production-related phenotype. For example, Jersey cows display a lower total amount of milk production but higher milk protein and fat content compared to those of Holstein cows [16]. Their phenotype differences affect the high metabolic heat production associated with rumen fermentation and lactation, and ultimately, it may contribute to sensitivity to heat stress [17].

It is well known that milk yield in *Bos taurus* dairy breeds begins to decline at temperature-humidity index (THI) 72, and at THI 68 for high-producing cows (35 kg/d) [5,18,19,20]. Despite the THI stress thresholds, variation among breeds in relation to milk production to heat stress conditions has been reported [21,22,23]. An early study suggests that the milk yield of Holstein and Jersey cows at a temperature of 29 °C and 40% relative humidity was 97% and 93% of normal, but when relative humidity was increased to 90%, it was 65% and 75% of normal [24]. In the study of the THI stress thresholds, it is reported that a decline in milk by the Holstein breed was more rapid than the Jersey breed across a range of THI from 72 to 84 [3]. Many other studies also reported that Jersey cows are more acclimated to hot and humid environments than Holstein cows, with constant milk yield and quality at a hot temperature [21,22,25]. Genetic differences between these two breeds may be unable to explain their physiological differences, suggesting that other factors may contribute to the differences. One potential explanation may be differences in the rumen microbiome. It is noticeable that heat stress conditions alter the rumen microbiome [26]. The rumen harbors highly dense and diverse microbial populations that play important roles in metabolism and health. The structure and function of the microbial community within the rumen is shaped by physical and chemical factors [27], such as diet [28], feeding program [29], environment [30], feeding behavior [31], and animal factors [32]. Despite the importance of genotype in determining the microbial community composition and enhancing the immune-related phenotype [33], only limited information on the comparison of the overall microbial compositions of different breeds of dairy cattle is currently available.

As the ruminal microbiome is an essential factor in understanding changes in the physiology and productivity of dairy cows, our study was designed to analyze changes in the rumen microbiome in the Holstein and Jersey breeds of dairy cows after exposure to heat stress. We hypothesize that ambient temperature differentially alters the rumen microbiome, which is associated with resistance against the heat stress response in different breeds of dairy cows. The aim of this study is to understand the potential contribution of the rumen microbiome to heat stress resistance in dairy cows.

## 2. Materials and Methods

Animal care and experimental procedures were conducted according to the guidelines of the National Institute of Animal Science (NIAS; Rural Development Administration, Cheonan 31000, Republic of Korea), Animal Care and Use Committee (Approval number: NIAS-2019107).

### 2.1. Animals and Diet

Before the beginning of the trial, the sample size was calculated using G*Power (Version 3.1.9.4 for Windows 1992–2019, Universität Kiel, Kiel, Germany). Repeated measures were calculated and considered optimal for a significance level of 0.05, a power of 0.8, and an effect size of 1.0. Based on the power test, a minimum number of 16 cows were required as the total sample size. Statistical power tests followed the guidelines suggested by Cohen in the field of animal science [34]. Holstein (60 ± 10.2-months-old; body weight, 715 ± 54.4 kg; n = 8) and Jersey cows (57 ± 9.3-months-old; body weight, 538 ± 20.3 kg; n = 8) were used for the experiments. Holstein cows averaged a milk yield of 39.3 ± 3.09 kg/d (139 ± 8.0 days in milk (DIM)) in May and 31.3 ± 4.67 kg/d (234 ± 8.0 DIM) in August, and Jersey cows averaged a milk yield of 31.0 ± 4.45 kg/d (128 ± 15 DIM) in May and 25.2 ± 5.6 kg/d (223 ± 15 DIM) in August. All Holstein cows had been a part of the National Institute of Animal Science (NIAS) dairy herd throughout their lives, whereas the Jersey cows were born through embryo transfer in the uterus of Holstein cows and had been part of the NIAS dairy herd for over 40 months under the same management. Cows were housed in individual tie stalls and were fed the same diet twice daily. The barn was equipped with a fan system, which was turned on at 09:00 and turned off at 18:00. The diets of cows were formulated to meet or exceed the energy requirements and fed ad libitum as a total mixed ration (TMR) to avoid the selection of dietary components. A total of 20 kg of feed was offered at 09:00 and 16:00 daily to each cow. There were no orts from any of the cows. The ingredients and nutrients of the experimental diet are shown in Table 1.

### 2.2. Measurement of Temperature–Humidity Index (THI) and Heat Stress

To determine the effects of heat stress on the ruminal microbiome, two distinct time points, as normal and heat stress conditions, were determined based on THI. For THI, temperature and humidity were determined using a thermos and humidity meter (Testo 174H, Testo Korea Ltd., Republic of Korea). The THI equation used was devised by the National Research Council (NRC) [35], and the equation is as follows: THI = (1.8 × ambient temperature + 32) − ((0.55 − 0.0055 × relative humidity) × (1.8 × ambient temperature − 26)). The THI on 3 May, as a normal condition, was 69.6 (24.4 °C and 36.3% rH), and on 6 August, as a heat stress condition, was 87.5 (35.5 °C and 59.6% rH). Rectal temperature and respiration rate were used to determine the degree of heat stress and were both measured at 14:00. Respiration rates were measured by counting flank shifts during a 15-s interval and multiplied by 4 to obtain breaths per minute. Rectal temperatures were measured using a standard digital thermometer (KD-133, Polygreen Co., Ltd., Stahnsdorf, Germany). To reduce deviating measures, the average value was used by measuring each cow twice.

### 2.3. Rumen Content Sampling

Rumen fluid samples were collected 5 h after morning feeding via stomach tubing in all Holstein and Jersey cows, once per cow [36], at the two timepoints (3 May, as a normal condition, and 6 August, as a heat stress condition) immediately after measuring rectal temperature and respiration rates. The first 150 mL of rumen fluid was discarded to minimize saliva contamination. Then, 45 mL of rumen fluid was collected and placed into a 50-mL conical tube (Thermo Scientific Inc., Waltham, MA, USA). All samples were stored at −80 °C in a freezer until use for DNA extraction and microbial community analysis.

### 2.4. DNA Extraction and Purification

DNA was extracted using the PowerSoil^®^ DNA Isolation Kit (Cat. No. 12888, MO BIO) according to the manufacturer’s protocol. Bead beating (0.1-mm glass beads; Sigma-Aldrich, St. Louis, MO, USA; bead beater, Bullet Blender Storm 24, Averill Park, NY, USA) at a speed of 4000 rpm for 30 s was used to homogenize the suspension and mechanically disrupt the bacterial cells. The purity and concentration of genomic DNA were measured using a spectrophotometer (Nanodrop 115 1000, Thermo Fisher Scientific Oxoid Ltd., Basingstoke, UK), and the integrity of DNA was verified by agarose (0.7%) gel electrophoresis. DNA samples were stored at −80 °C until further processing. The extracted DNA was further sequenced, processed, and analyzed by Macrogen (Macrogen Inc., Seoul, Korea).

### 2.5. 16S rRNA Gene Sequencing and Analysis

The V3–V4 regions of the 16S rRNA gene were amplified by PCR using primers that contained an ILMN preadapter + sequencing primer + specific locus primer: V3 (5′-TCGTCGGCAGCGTC + AGATGTGTATAAGAGACAG + CCTACGGGNGGCWGCAG-3′; forward) and V4 (5′-GTCTCGTGGGCTCGGA + GATGTGTATAAGAGACAGG + ACTACHVGGGTATCTAATCC-3′; reverse). Equimolar amounts of the barcoded V3–V4 amplicons were pooled and paired-end sequenced on an Illumina MiSeq PE300 platform (Illumina, Inc., San Diego, CA, USA). Raw sequences were trimmed (Trimmomatic v0.38) [37] and filtered using FLASH (v. 1.2.11) [38]. To ensure that any subsequent analysis was highly accurate, sequences shorter than 400 bp were discarded. To avoid biases generated by differences in sequencing depth, each sample was subsampled to an even depth of 10,000 reads. The filtered reads were clustered and identified as operational taxonomic unit (OTU) sequences at 97% similarity using CD-HIT-OTU (http://weizgongli-lab.org/cd-hit-otu/), and chimeric sequences were identified and removed using rDnaTools (https://github.com/PacificBiosciences/rDnaTool). Sequences were classified using the Ribosomal Database Project (https://github.com/PacificBiosciences/rDnaTool). Community diversity was estimated using the Shannon and Simpson indices [39,40]. The unweighted UniFrac distance method was used to perform a principal coordinates analysis, and analysis of molecular variance was conducted to assess significant differences among samples using the program MOTHUR (v. 1.35.1).

### 2.6. Whole-Genome Shotgun Sequencing and Analysis

The extracted DNA was quantified using the TruSeq Nano DNA library prep guide (Illumina), and then a DNA library was constructed using an LE220 focused-ultrasonicator (Covaris, Inc., Woburn, MA, USA). Thereafter, the DNA library was concentrated, purified, and sequenced using the HiSeq™ 4000 platform (Illumina, San Diego, CA, USA). After sequencing, the raw sequence data files were demultiplexed and stored in the fastq format. The sequence data were assembled using an IDBA-UD assembler [41]. FastQC (FastQC; ver. 0.11.8) was used for checking the quality score of raw reads, and KneadData (KneadData; The Huttenhower Lab; http://huttenhower.sph.harvard.edu/kneaddata) was used to improve the accuracy of the analysis; data analysis was performed after removing the adapter sequence. For gene prediction, contigs were assembled and annotated using Prodigal v.2.6 software [42] in the metagenomic mode, and the predicted genes were then annotated using the eggNOG database [43] with a BLAST search (E-value ≤ 1.0 × 10^−5^) and classified according to their functions. The obtained reads were taxonomically classified into the groups using the centrifuge method [44], and the reads of each sample were analyzed using the NCBI database to determine breed information by group. The linear discriminant analysis effect size (LEfSe) method [45], based on the Kruskal–Wallis (KW) sum–rank test, was performed to identify the difference in microbial populations in each group to characterize the microbial populations, from phylum to breed level, that were differentially abundant in the high-temperature stress condition. For this analysis, the critical threshold of the KW test was set to 0.05, and the algebraic linear discriminant analysis score [46] was set to ≥3.0. The set of protein sequences of the predicted genes was aligned with the KEGG gene database [47,48,49] using BLAST (E-value ≤ 1.0 × 10^−5^) to obtain functional annotation information. To confirm the quantitative difference of genes related to heat stress between samples, the bacterial heat shock protein (HSP) protein sequence registered in UniProt [50,51] was obtained and analyzed against the genetic information of each sample.

### 2.7. Statistical Analyses

All statistical analyses were performed in SAS 9.4 (SAS version 9.4; SAS Institute Inc., Cary, NC, USA). For individual data on heat stress responses (respiration rate and rectal temperature), the relative abundance of KEGG genes, nutrient metabolism (energy, amino acid, and carbohydrate) genes, HSP-related genes, and the relative abundance of predicted protein domains and biodiversity (Shannon and Simpson indices) were analyzed using a PROC MIXED model for repeated measures. The statistical model included the fixed effect of two breeds (Breed; Holstein or Jersey cows), two timepoints (Time; normal or heat stress conditions and the interaction of two factors (Breed × Time), and random effects of cows within a breed. Differences between least-square means for fixed effects were estimated with Tukey’s adjustment option. Significant differences were declared at *p* ≤ 0.05.

## 3. Results

### 3.1. Heat Stress Response Differences between Holstein and Jersey Cows in Hot Weather Condition

**Figure 1:**
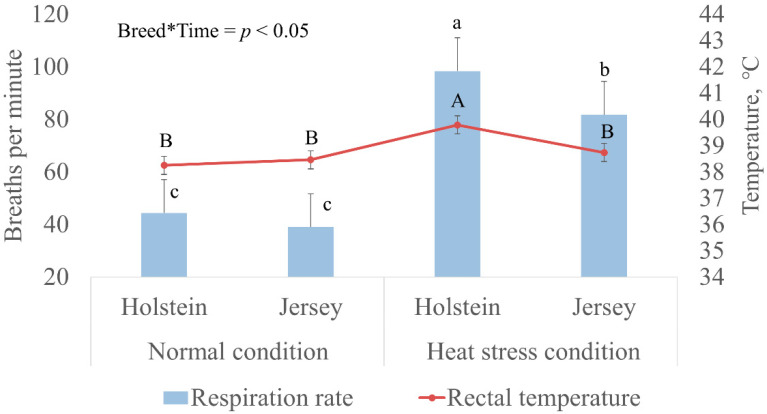
Holstein and Jersey cows display differential heat stress responses at the ambient temperature. Temperature–humidity index (THI) was measured in May, as the normal environment, and in August, as the heat stress environment, and were 69.6 and 87.5, respectively. Bar (a,b) or graph (A,B) with different letters differ significantly (*p* < 0.05).

In this study, we compared the respiration rate and rectal temperature between Holstein and Jersey cows in May, as the normal condition, and in August, as the heat stress condition. The respiration rate and rectal temperature were not different between Holstein and Jersey cows in normal conditions (Figure 1). However, the two breeds showed differential stress responses in heat stress conditions. Although the respiration rate in both breeds increased (*p* < 0.05) in heat stress conditions (THI 87.5) compared to that in normal conditions (THI 69.6), Holstein cows (98.4 breaths/min) had a higher respiration rate compared to that of Jersey cows (81.8 breaths/min) in heat stress conditions. In addition, heat stress increased the rectal temperature in Holstein cows but not in Jersey cows (*p* < 0.05; 39.8 vs. 38.7 °C, respectively). These results indicated that Holstein cows are more susceptible to ambient temperature with prominent heat stress responses. This allowed us to compare the ruminal microbiome between Holstein and Jersey cows in the ambient temperature.

### 3.2. Changes in the Ruminal Microbial Diversity of Holstein and Jersey Cows in the Heat Stress Environment

**Figure 2:**
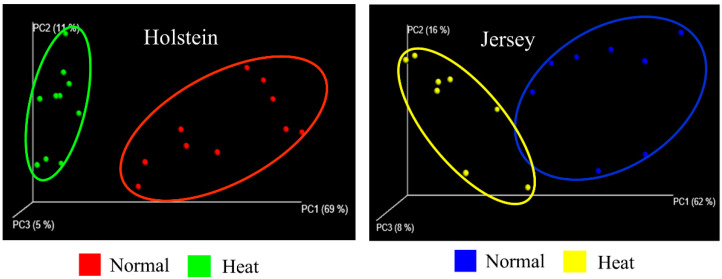
Effect of ambient temperature on ruminal microbial diversity in Jersey and Holstein cows. Unweighted UniFrac principal coordinates analysis (PCoA) profiles of ruminal microbial diversity were determined for ruminal microbes.

Sequencing of the 16S rRNA genes in the rumen fluid produced a total of 170,322 reads, which were rarefied across samples to the lowest sample depth (data not shown). First, we examined the diversity of the microbial communities in the rumen of Holstein and Jersey cows in normal and heat stress conditions. Unweighted UniFrac principal coordinates (PCoA) plot analysis showed a clear separation of microbial diversity by heat stress between Holstein and Jersey cows (Figure 2). The diversity of the microbial communities in the rumen samples was measured using Shannon and Simpson indices [39,40]. Microbial community richness and evenness, as represented by Shannon (≥7.48) and Simpson (≥0.974) diversity indices, showed high microbial diversities in both conditions regardless of breed (Table 2). The unweighted UniFrac PCoA plot indicated that there was no significant difference between Holstein and Jersey cows in both conditions (Figure S1). These results indicate that the heat stress environment affects ruminal microbial diversity in both breeds in an independent manner.

### 3.3. Taxonomic Classification of the Ruminal Bacteria

**Figure 3:**
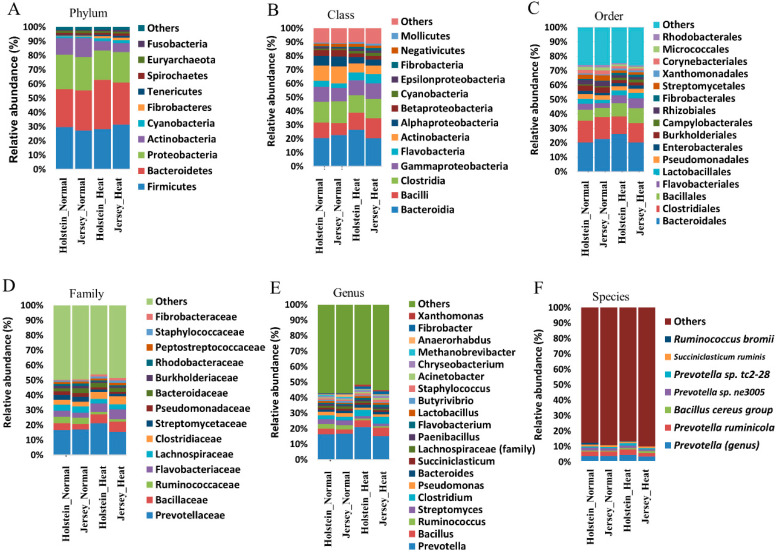
Impact of heat stress on the ruminal bacterial composition of Holstein and Jersey cows. Taxonomic classification of the 16S rRNA gene sequence at the (**A**) phylum, (**B**) class, (**C**) order, (**D**) family, (**E**) genus, and (**F**) species levels for Holstein and Jersey cows in normal and heat stress conditions.

We performed a taxonomic classification of the ruminal bacteria to determine whether heat stress affects ruminal microbiota composition. Comparisons of the relative abundance of the ruminal microbiota compositions among four groups at the phylum, class, order, family, genus, and species levels are shown in Figure 3A–F. The taxonomic analysis of the reads revealed the presence of four predominant phyla (average relative abundance ≥ 2%) in the rumen in the normal condition, regardless of the breed (Figure 3A): *Firmicutes* (29.6% in Holstein cows and 27.3% in Jersey cows), *Bacteroidetes* (26.7% and 28.1% in Holstein and Jersey cows, respectively), *Proteobacteria* (24.2% and 23.6% in Holstein and Jersey cows, respectively), and *Actinobacteria* (11.7% and 13.2% in Holstein and Jersey cows, respectively). At the genus level, 42.9% of the total reads were annotated, and taxonomic analysis revealed the presence of six predominant genera (average relative abundance ≥ 2%) in the rumen of Holstein and Jersey cows in the normal condition: *Prevotella* (16.3% and 16.7% in Holstein and Jersey cows, respectively), *Bacillus* (3.6% and 2.7% in Holstein and Jersey cows, respectively), *Ruminococcus* (3.0% and 2.8% in Holstein and Jersey cows, respectively), *Streptomyces* (3.0% and 3.3% in Holstein and Jersey cows, respectively), *Clostridium* (2.7% and 2.2% in Holstein and Jersey cows, respectively), and *Pseudomonas* (2.3% and 2.3% in Holstein and Jersey cows, respectively) (Figure 3E). The overlapping ruminal bacteria counts by the taxonomic classification are shown in Figure S2. The counts of shared ruminal bacteria among four groups at the family, genus, and species level were 547, 3366, and 23,093 counts, respectively.

**Figure 4:**
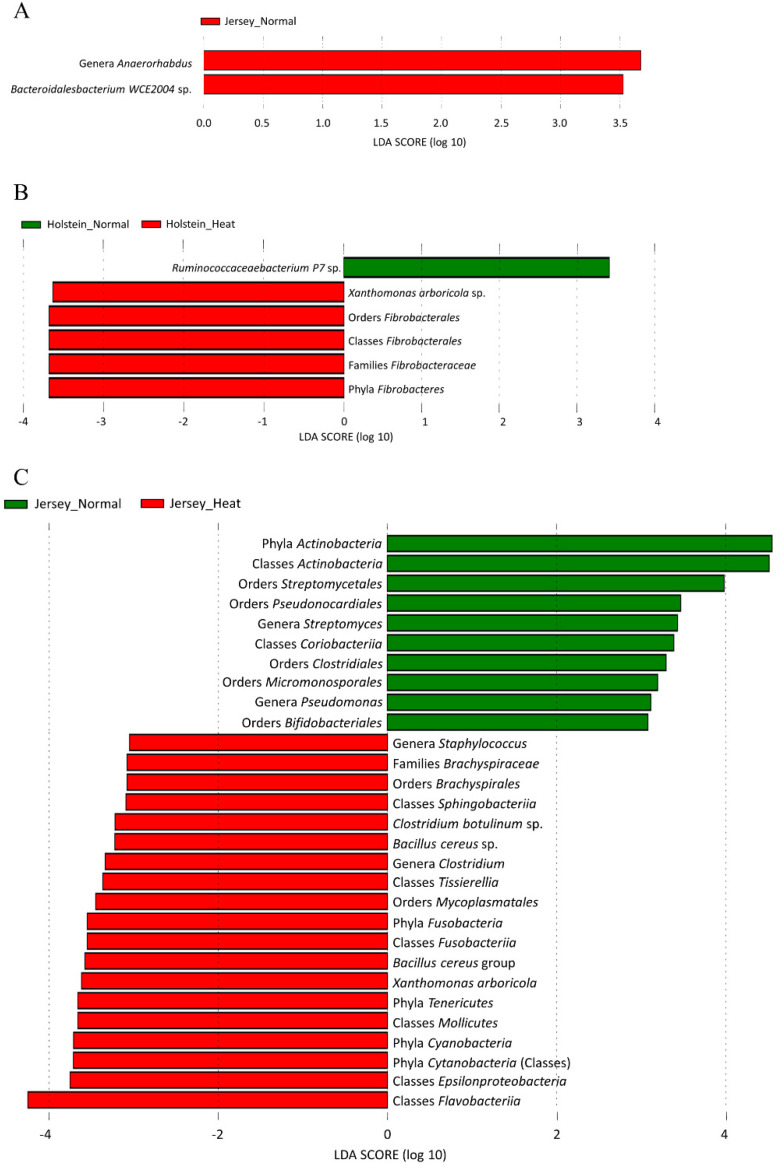
Linear discriminant analysis effect size (LEfSe) plots of differentially abundant rumen bacterial taxa. (**A**) Taxonomic comparison of ruminal bacteria in Jersey cows enriched either in the normal or heat stress condition. (**B**,**C**) Taxonomic comparison of ruminal bacteria in Holstein (**B**) and Jersey cows (**C**) between normal and heat stress conditions.

We further analyzed the relative abundance among taxonomically classified bacteria to determine the singular effect of heat stress in the rumen microbiota of the breeds by LEfSe. First, we determined the effects on the breeds in the same condition, i.e., either normal or heat stress. This analysis revealed that the genera *Anaerorhabdus* and *Bacteroidalesbacterium WCE2004* sp. were the taxa distinguishing Jersey cows from Holstein cows in the normal condition, whereas there was no difference between the breeds in the heat stress condition (Figure 4A). Next, we determined the effects of heat stress in each breed. In Holstein cows, six taxa were identified as distinguishing taxa between the normal and heat stress condition (Figure 4B). *Ruminococcaceae bacterium P7* sp. was the only enriched taxon in the normal condition, whereas five taxa, i.e., phylum *Fibrobacteres*, class *Fibrobacteria*, order *Fibrobacterales*, family *Fibrobacteraceae*, and *Arboricola* sp., were enriched in the heat stress condition compared with the normal condition (Figure 4B). Jersey cows, in the normal condition, had an enriched relative abundance of the phylum *Actinobacteria*, followed by the class *Actinobacteria* and *Coriobacteriia*, orders *Streptomycetales*, *Pseudonocardiales*, *Clostridiales*, *Micromonosporales*, and *Bifidobacteriales*, and genera *Streptomyces* and *Pseudomonas* compared to those in Jersey cows in the heat stress condition (Figure 4C). Moreover, the phyla *Fusobacteria*, *Tenericutes*, and *Cyanobacteria*; *Sphingobacteria*, *Tissierella*, *Fusobacteria*, *Mollicutes*, *Epsilonproteobacteria*, and *Flavobacteria*; *Brachyspirales* and *Mycoplasmatales*; one taxon of the family *Brachyspiraceae*; genera *Staphylococcus* and *Clostridium*; *C. botulinum* sp., *B. cereus* sp., *B. cereus group* sp., and *Xanthomonas arboricola* sp. were enriched in Jersey cows in the heat stress condition (Figure 4C). In general, changes of different magnitudes and contexts in bacterial composition were observed between Holstein and Jersey cows, and Jersey cows showed more changes in the ruminal microbiome in response to heat stress. Collectively, we did not observe significant changes in the major population at each level, but there were differential changes in minor bacterial taxa in our study.

### 3.4. Microbial Function Characteristics of Ruminal Metagenomics

We assessed the enrichment of functional genes in the ruminal microbiome by whole metagenomic sequencing using the HiSeq Illumina platform. Domains predicted by the distribution of clusters of orthologous groups (COGs) in the rumen sample using the eggNOG database are shown in Figure S3. We found that some predicted protein domains were significantly enriched in specific groups. For example, genes associated with energy production and conversion were enriched in Jersey cows in the normal condition, and heat stress decreased the relative proportion of these genes in Jersey cows (*p* < 0.05; Figure S4). Genes associated with the defense mechanism, lipid transport and metabolism, coenzyme transport and metabolism, cell cycle control, cell division, and chromosome partitioning increased (*p* < 0.05) in Holstein cows by heat stress, but Jersey cows had similar levels of relative abundance in normal and heat stress conditions. The relative abundance of genes associated with cell motility and extracellular structures decreased by heat stress only in Holstein cows.

**Figure 5:**
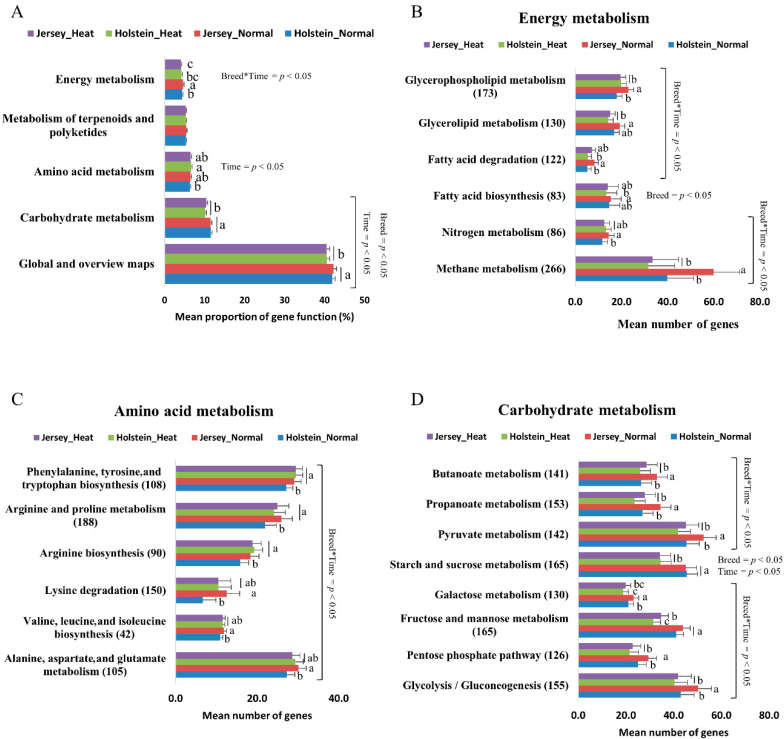
Variations in KEGG metabolic pathways by functional bacterial communities. (**A**) Comparisons of the five predominant gene pathways of the ruminal microbiome. (**B**–**D**) Comparison of the functional capacities of the ruminal microbiomes associated with nutrient metabolism between Holstein and Jersey cows. Normalized abundance of the level 3 SEED subsystem classified reads associated with energy (**B**), amino acid (**C**), and carbohydrate (**D**) metabolism.

The analysis of the second level of KEGG pathways revealed that the proportions of gene functions in global and overview maps and carbohydrate metabolism were different in both breeds between normal and heat stress conditions, but there was no impact of breed (Figure 5A). Genes associated with amino acid metabolism increased in Holstein cows by heat stress, but this was not the case in Jersey cows. Jersey cows had a higher proportion of genes associated with energy metabolism in normal conditions compared with that in Holstein cows; this difference was compromised in the heat stress condition by the decreased expression of such genes in Jersey cows.

We selected the third level KEGG pathways, which may be essential for heat production by ruminal fermentation (Figure 5B–D). For energy metabolism, Jersey cows, in normal conditions, had a higher abundance of genes associated with methane metabolism, nitrogen metabolism, fatty acid degradation, and glycerophospholipid metabolism compared with Holstein cows in the normal condition (Figure 5B). Heat stress decreased the number of these genes in Jersey cows, resulting in similar gene numbers between Holstein and Jersey cows. In addition, heat stress decreased gene numbers involved in glycerolipid metabolism in only Jersey cows (Figure 5B). In general, Jersey cows had more genes for energy metabolism-associated pathways, and the abundance of these genes was actively changed by heat stress compared with that in Holstein cows.

Jersey cows had a higher number of genes involved in all amino acid metabolism-associated pathways compared with that in Holstein cows in the normal condition (Figure 5C). However, heat stress affected amino acid metabolism-related gene abundance. In the normal condition, Jersey cows had a higher number of genes for alanine, aspartate, and glutamate metabolism and valine and lysine degradation than Holstein cows. However, heat stress altered these gene numbers, leading to a similar number of genes in Holstein and Jersey cows. Additionally, heat stress elevated the number of genes for arginine biosynthesis, arginine and proline metabolism, and phenylalanine, tyrosine, and tryptophan biosynthesis in Holstein cows but not in Jersey cows (Figure 5C). In general, Jersey cows had more genes for amino acid metabolism-associated pathways, and the abundance of these genes was increased in Holstein cows by heat stress.

In relation to carbohydrate metabolism, Jersey cows in the normal environment had a higher number of genes for glycolysis/gluconeogenesis, the pentose phosphate pathway, galactose metabolism, pyruvate metabolism, propanoate metabolism, and butanoate metabolism (Figure 5D). The number of genes associated with these functional metabolisms was decreased in only Jersey cows, except for galactose metabolism, which decreased in both breeds. The reduction of these genes by heat stress led to similar gene numbers between Holstein and Jersey cows in the heat stress condition. The number of genes associated with fructose and mannose metabolism and starch and sucrose metabolism was similar between both breeds in normal conditions and decreased by heat stress in both Holstein and Jersey cows (Figure 5D). In general, Jersey cows had more genes for carbohydrate metabolism-associated pathways, and a reduced abundance of these genes by heat stress was more prominent in Jersey cows. Collectively, Jersey cows had a higher number of genes associated with energy, amino acid, and carbohydrate metabolism in the normal condition. The abundance of genes for energy and carbohydrate metabolism was more clearly decreased by heat stress in Jersey cows, whereas the abundance of genes for amino acid metabolism was more clearly increased by heat stress in Holstein cows.

**Figure 6:**
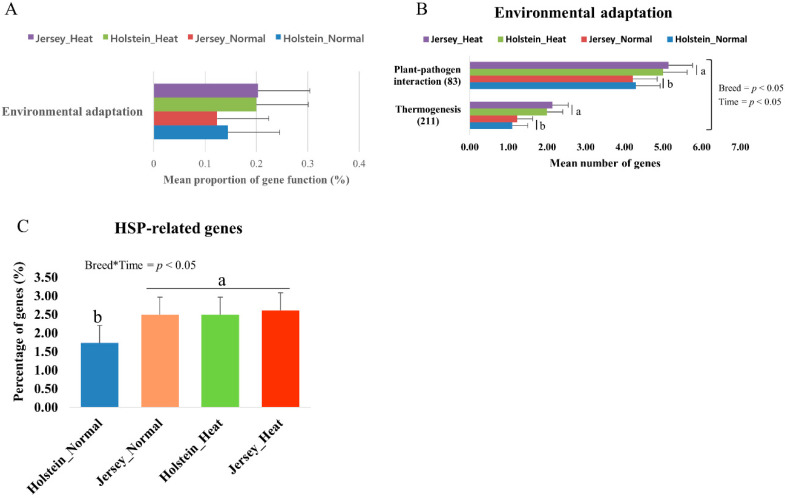
Comparison of the abundances of environmental adaptation and heat shock protein (HSP) genes by functional bacterial communities. (**A**) Comparisons of the abundances of gene pathways of the ruminal microbiome for environmental adaptation. Comparison of the functional capacities of the ruminal microbiomes associated with nutrient metabolism between Holstein and Jersey cows. Normalized abundance of the level 3 SEED subsystem classified reads associated with environmental adaptation (**B**). (**C**) Comparison of the percentage of HSP-related genes in rumen samples associated with heat stress. Bars with different letters (a, b) differ significantly (*p* < 0.05).

The heat stress response also affected environmental adaptation pathways. In this study, heat stress increased (*p* < 0.05) the proportion of genes for environmental adaptation, regardless of the breed (Figure 6A). In the analysis for pathways in KEGG, we observed that the number of genes for thermogenesis and plant–pathogen interaction was increased by heat stress, but there were no significant differences between Holstein and Jersey cows in different conditions (Figure 6B). The relative abundance of HSP-related genes was higher in Jersey cows than that in Holstein cows in the normal condition (Figure 6C). Heat stress increased HSP-related genes in Holstein cows but not in Jersey cows. In summary, heat stress increased the number of genes for environmental adaptation in the rumen of dairy cows; however, there were no such differences between Holstein and Jersey cows.

## 4. Discussion

We performed this study to better understand the potential nonanimal genetic factors that affect the heat stress resistance of dairy cows. To this end, we designed an experiment to assess the changes in rumen microbiome in response to heat stress between Holstein and Jersey cows, as these two breeds display differential heat stress resistance. Physical muscle activity increases heat production by the body by increasing the respiration rate; thus, the respiration rate is well-known as a part of the mechanism of the body to release heat. Therefore, the respiration rate has been used as an indicator of the body’s physiological response to heat stress, together with rectal temperature, in cattle [52,53]. The THI has been used as an index for the environmental conditions [54]. It has been reported that the rectal temperature of cattle is positively correlated with THI. For example, in Holstein cows, in the normal condition, the rectal temperature range was 38.4–38.8 °C when THI ranged from 60 to 72 [55,56]. In contrast, in the heat stress condition, with a THI range of 72–89, the rectal temperature range was 39.0–39.1 °C [57,58]. The rectal temperature of Jersey cows is consistently lower than that of Holstein cows under the same conditions without solar radiation [21]. For example, Jersey cows display a mild heat stress response, with rectal temperatures ranging from 37.2 to 38.7 °C in a heat stress environment (THI 70–80) [59]. In general, Jersey cows showed lower rectal temperatures across the same THI range compared with those of Holstein cows, which have a 0.3 °C higher rectal temperature than that of Jersey cows. In addition, a decline in milk yield in Holstein cows in ambient temperature is more rapid than that in Jersey cows, with a THI range from 72 to 84 [3]. In our study, the THI values were 69.6 and 87.5 in the normal and heat stress conditions, respectively. Based on the assessment of the respiration rate and rectal temperature, Holstein and Jersey cows showed distinct heat stress responses (Figure 1). Distinct THI and heat stress responses of the two breeds suggest that our study was properly performed to determine the differential changes in the rumen microbiome between Holstein and Jersey cows during heat stress.

It has been suggested that diet [28], feeding strategy [29], environment [30], feeding behavior [31], and animal factors [32] have impacts on the microbial composition and stability of microbial ecosystems in the rumen. The heat stress response affects the feed intake of dairy cows [3,6]. Although we fed the same TMR diet ad libitum to both Holstein and Jersey cows, we did not observe any limitation in diet intake in either breed during this study, suggesting that some observed rumen microbiome changes cannot be driven by selective diet intake in a heat stress environment.

Traditionally, bacterial diversity has been used to determine the effect of environmental factors, such as selective consumption of diet, on the rumen microbiome. Shannon and Simpson indices have been used as representative indicators for microbial diversity [60]. Shannon and Simpson indices indicate higher weights of bacterial richness and uniformity, respectively [39,40]. In the current study, the PCoA plots indicated that Holstein and Jersey cows had a relatively clear separation of microbial diversity under heat stress (Figure 2). However, there were no significant differences in Shannon and Simpson indices. Taxonomic bacterial compositions showed a similarity of predominant bacterial phyla between Holstein and Jersey cows (Figure 3). In addition, several studies have reported that diet composition and selective consumption highly influence ruminal bacterial diversity [61,62]; particularly, *Firmicutes* is a major phylum that can be altered by diet [63,64,65]. However, we did not observe significant changes in the phylum *Firmicutes* and other major bacterial phyla in our study. Therefore, we assume that certain changes in the rumen microbiome may not be fully explained by differential or selective diet intake by Holstein and Jersey cows.

Interestingly, we observed differential changing patterns of the rumen microbiome between Holstein and Jersey cows under heat stress. In the assessment of the rumen’s bacterial composition by 16S rRNA sequencing, heat stress conditions significantly increased the relative abundance of the phylum *Fibrobacteres*, class/order *Fibrobacterales*, and family *Fibrobacteraceae* only in the rumen of Holstein cows (Figure 4A). One possible explanation for this is that the Fibrobacteres group (phylum to order) has stronger heat resistance than other ruminal bacteria. In general, all microorganisms have specific favorable temperature conditions and heat resistance; therefore, each ruminal microbe proliferates differentially at certain rumen temperatures [66]. In this regard, an increase in rectal temperature in Holstein cows may provide a favorable environment for the *Fibrobacteres* group. The *Fibrobacteres* group (phylum to family) is linked to cellulolytic activity. Cellulolytic activity, as a typical metabolism of rumen microbes, enables harvesting of energy from the diet [67,68]. The efficiency of rumen microbial metabolism is closely related to heat generation in the rumen, and heat generation can be indirectly estimated through the efficiency of bacterial growth [69]. The metabolism of microbes is inefficient when they do not direct all ATP toward their growth, as microbes utilize some ATP for maintenance functions, synthesis of reserve carbohydrates, and energy spilling [70,71]. This inefficient metabolism occurs when the diet is limited, resulting in energy loss by methane production in the rumen. Webster [72] reported that based on the ratio of energy conversion to the final product of rumen fermentation, energy production with acetate was three times higher than that with propionate, and 15% of energy was lost to methane when animals were fed only hay. Currently, we do not fully understand the interaction between efficiency of microbial metabolism, including methane production and heat production, in the rumen. However, there is the possibility that inefficient metabolic responses by rumen bacteria may exaggerate heat production in Holstein cows in the heat stress condition. In addition, the relationship between ruminal heat production and specific microbes under heat stress conditions has not been clearly investigated. In our study, we could not conclude that the increased *Fibrobacteres* count observed contributes to heat production in Holstein cows at the ambient temperature due to a lack of available information. Acetate produces more ATP than other volatile fatty acid metabolisms [73]. *Fibrobacteres* are well-known to degrade plant-based cellulose in ruminants and produce acetate through fiber fermentation [74]. Furthermore, Ransom-Jones and colleagues [75] reported that glycosyl hydrolases of the phylum *Fibrobacteres* have the potential to produce carbohydrate activators, including cellulose enzymes, suggesting that Holstein cows may ultimately produce more energy with acetate in the rumen and/or energy spilling, and this particular energy metabolism may be associated with heat production in Holstein cows.

It is worth noting that Jersey cows showed a differential changing pattern in bacterial species under heat stress compared with Holstein cows. In Jersey cows, the abundance of the Actinobacteria group (from phylum to genus) decreased by heat stress (Figure 4C). *Actinobacteria* are classified as Gram-positive bacteria and have extensive capabilities to metabolize diet, in particular starch and starch-like polysaccharides and oligosaccharides, producing lactic and acetic acids as the main metabolic end-products [76]. In contrast, the abundances of rumen bacteria involved in fiber degradations, such as phylum *Fusobacteria* and *Cyanobacteria*, which are unable to process cellulose [77,78], increased in Jersey cows in the heat stress condition. Interestingly, we could not observe these changes in Holstein cows in the heat stress condition. Collectively, differential enrichment of rumen bacteria by heat stress between Holstein and Jersey cows suggests that rumen microbiome in Jersey cows may adapt differently and/or change in response to heat stress compared to those in Holstein cows. There is another possibility that the increased temperature of the rumen environment due to the ambient temperature induces a selection of the rumen bacteria that results in reducing heat resistance in dairy cows; rumen bacteria in Holstein and Jersey may undergo a different selection process in response to heat stress.

As mentioned earlier, however, there is no evidence for the relationship between heat response and a specific rumen bacterial population. When we consider that Jersey cows are better able to cope with heat stress conditions than Holstein cows, a possible assumption is that acetic-acid-producing bacteria such as the *Actinobacteria* group or *Fibrobacteres* may contribute to heat stress responses in dairy cows by increasing heat production in the rumen metabolism. As limited information on heat production by specific rumen microbes is available, future studies need to be undertaken to determine whether ambient temperature induces the selection of the rumen bacteria and to understand the role of the rumen bacteria changes in the regulation of heat resistance.

Although we observed differences in bacterial taxa in minor groups, there is still the possibility that the abundance of functional genes of the ruminal microbiome can be altered by heat stress. Several studies have emphasized that exposure to heat stress conditions results in the downregulation of metabolic pathways in the rumen [26,79,80]. Moreover, heat stress increases the abundance of genes for environmental adaptation [26]. In agreement with previous studies, in both breeds, genes for environmental adaptation were upregulated, but genes for metabolism were downregulated by heat stress in our study. It is worth noting that Jersey cows had a higher abundance of metabolism-related genes, including those related to energy, amino acid, and carbohydrate metabolism in the normal condition compared to Jersey cows in the heat stress condition. However, heat stress actively decreased the abundance of genes associated with energy and carbohydrate metabolism in only Jersey cows; thus, both breeds had a similar abundance level of genes for these metabolic pathways in the heat stress condition. Currently, we do not fully understand how reduced abundances of genes for energy, amino acid, and carbohydrate metabolism are associated with reduced heat stress responses in Jersey cows. However, one clear finding from this study is that rumen microbiota in Jersey cows more actively changes the abundance of microbial genes associated with rumen metabolism, unlike the microbiota in Holstein cows that undergoes no significant changes under heat stress. This finding is supported by the observation that the rumen microbial composition in Jersey cows changed more under heat stress. A more active and distinctly altered rumen microbiome in Jersey cows by heat stress may lead to enhanced heat stress resistance. Based on the differentially changing magnitude in the rumen microbiome, affecting rumen function [81], we conclude that the rumen microbiota of Jersey cows has a better capacity to adapt to environmental challenges, such as ambient temperature, and this is possibly attributed to active shifts in the rumen microbiome. However, we did not observe differences in genes for environmental adaptation. This may be due to a technical limitation; therefore, a more specific approach to determine the abundance of genes responsible for heat stress adaptation is needed.

We also further analyzed the abundance of HSP-related genes associated with the heat stress response and/or resistance [82,83]. Rosic and colleagues [84] reported that there are activation thresholds to the intensity of heat stress for each type of HSP, an important role of HSPs under diverse heat stress conditions in adapting to environmental changes, which indicate differences in HSP inducible expression. In addition, Richier et al. [85] suggested that environmental conditions may regulate the heat shock transcription factor for activation of heat-inducible genes and heat acclimation, and it is indicated by the difference in HSP expression. In our study, the abundance of HSPs was significantly higher in Jersey cows in the normal condition. However, heat stress increased the number of HSP genes in Holstein cows, resulting in similar abundance levels of HSPs in rumen microbes between Holstein and Jersey cows. This suggests that HSPs are not major players in the differential heat stress responses between Holstein and Jersey cows. Further studies are needed to elucidate the correlation between the abundance of HSP-related genes and heat stress in Jersey cows.

## 5. Conclusions

In Holstein cows, the composition of the distinct microbial community suggests that microbiome changes with the enrichment of Fibrobacteres under heat stress may be a source of heat generation. More prominent changes in rumen bacterial taxa and functional gene abundance in Jersey cows showed that they have a weak heat stress response in hot weather, suggesting that these changes likely help in adapting to climate change. The THI was not adequately applied for measuring heat stress intensity in the Jersey breed, suggesting that THI index adjustments or other indicators should be designed to measure cattle heat stress intensity. We believe that our results provide evidence for changes in the ruminal bacterial composition in heat stress response in both Holstein and Jersey dairy cow breeds. In addition, Holstein cows, which are vulnerable to heat, may help better understand changes in ruminal bacteria and genes for further studies aimed at developing ruminal bacterial regulators. The limitation of this study is that the correlation between production parameters, such as milk component, ruminal VFA, and body condition, and microbial communities, was not explored. Future studies are needed to clarify the correlation between rumen microbial changes and gene expression and productivity under heat stress.

## Figures and Tables

**Table 1:** Ingredients and nutrients of the experimental diets.

Item	Amount
Ingredients composition, % of DM	
Concentrate	15.3
Soybean meal	2.40
Corn silage	47.2
Alfalfa hay	7.10
Tall fescue	9.40
Timothy	5.90
Energy booster ^1^	7.10
Cash Gold ^1^	5.00
Lyzin-Plus ^2^	0.20
Limestone	0.20
Zin Care ^1^	0.10
Supex-F ^1^	0.50
Trace minerals ^3^	0.05
Vitamins premix ^4^	0.05
Chemical composition	
Dry matter (DM), %	53.2
Crude protein, % of DM	10.0
Neutral detergent fiber, % of DM	28.2
Acid detergent fiber, % of DM	16.9
Calcium, % of DM	0.40
Phosphorus, % of DM	0.15

^1^ Cofavet, Cheonan, Republic of Korea. ^2^ A.N. Tech, Cheonan, Republic of Korea. ^3^ Contained 0.40% Mg, 0.20% K, 4.00% S, 0.08% Na, 0.03% Cl, 400 mg of Fe/kg, 60,042 mg of Zn/kg, 16,125 mg of Cu/kg, and 42,375 mg of Mn/kg. ^4^ Provided approximately 5000 KIU of vitamin A/kg, 1000 KIU of vitamin D/kg, 33,500 mg of vitamin E/kg, and 2400 mg of vitamin C/kg.

**Table 2:** Biodiversity of ruminal microbiota using Shannon and Simpson indices.

Item	Normal Environment		Heat Stress Environment		SEM ^1^	*p*-Value		
	Holstein	Jersey	Holstein	Jersey		Breed	Time	Breed × Time
Shannon	7.48	7.76	7.83	7.95	0.410	0.16	0.06	0.54
Simpson	0.974	0.985	0.982	0.985	0.018	0.34	0.32	0.42

^1^ SEM, standard error of mean.

## Supplementary Materials

The following are available online at https://www.mdpi.com/2076-2615/10/7/1127/s1, Figure S1: Principal coordinates analysis (PCoA) and ruminal microbial diversity in Holstein and Jersey cows under normal and ambient temperature. Figure S2: Venn diagram illustrating the overlapping taxonomic classification of 16S rRNA gene at the (A) family, (B) genus, and (C) Species levels for Holstein and Jersey cows in normal and heat stress conditions. Figure S3: Clusters of orthologous groups (COGs) distribution of predicted protein domains in rumen samples associated with heat stress obtained from the eggNOG database. Figure S4: Variations in KEGG metabolic pathways in functional bacterial communities.
