# Supplementary material for: Differential Dynamics of the Ruminal Microbiome of Jersey Cows in a Heat Stress Environment
Source: Animals (Basel). 2020 Jul 2;10(7):1127. doi: 10.3390/ani10071127 (PMC7401637; doi:10.3390/ani10071127)
Supplement: Supplementary file 1 [file animals-10-01127-s001.pdf]

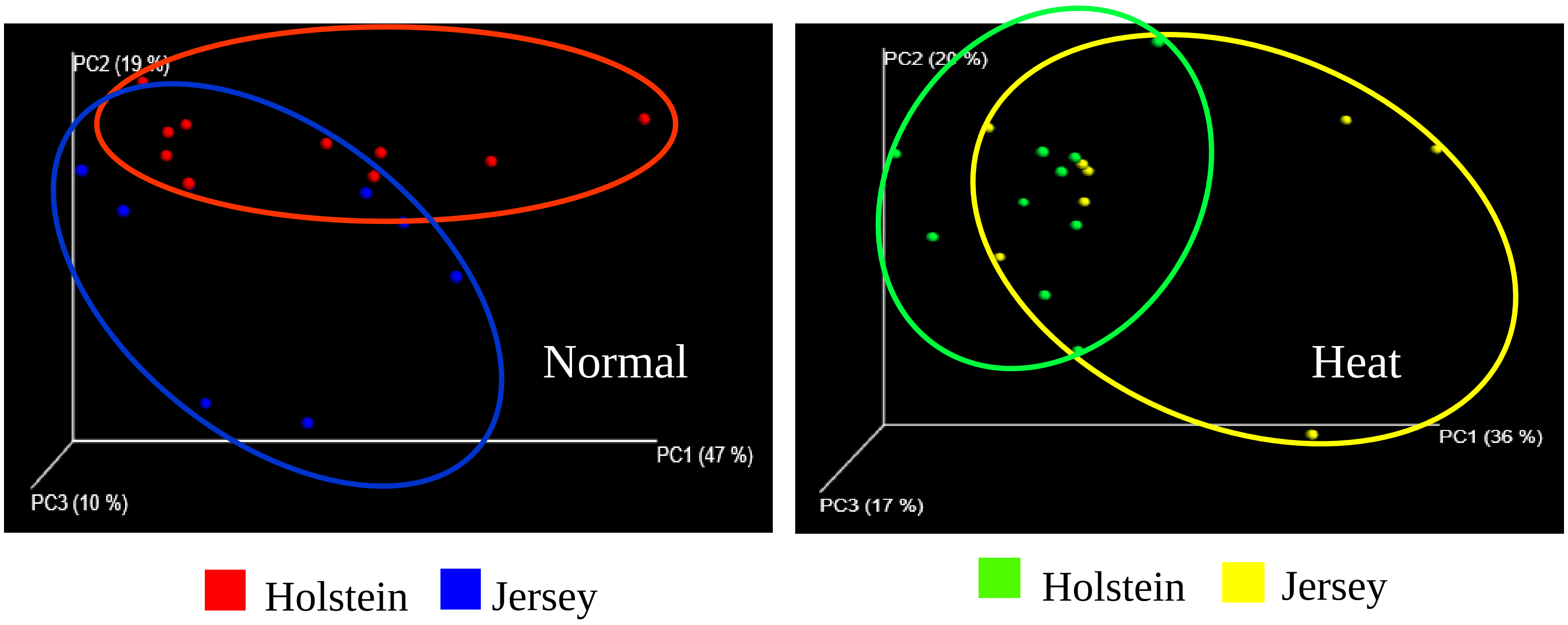

Figure S1. Principal coordinates analysis (PCoA) and ruminal microbial diversity for Holstein and Jersey cows in normal and ambient temperature. PCoA comparison between Holstein and Jersey cows' rumen samples collected under the normal and ambient temperature, respectively.

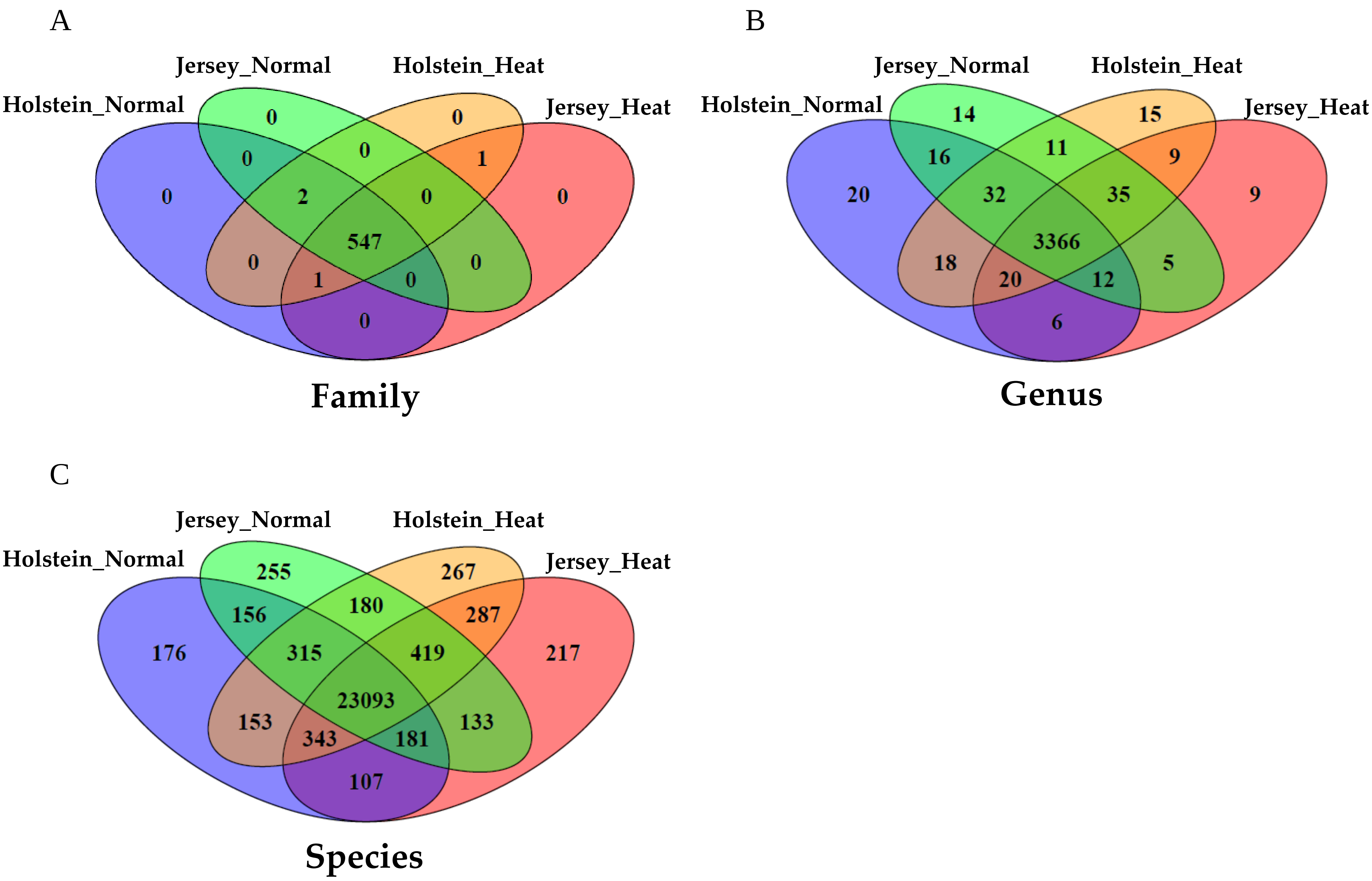

Figure S2. Venn diagram illustrating the overlapping taxonomic classification of 16S rRNA gene at the (A) family, (B) genus, and (C) Species levels for Holstein and Jersey cows in normal and heat stress conditions.

A

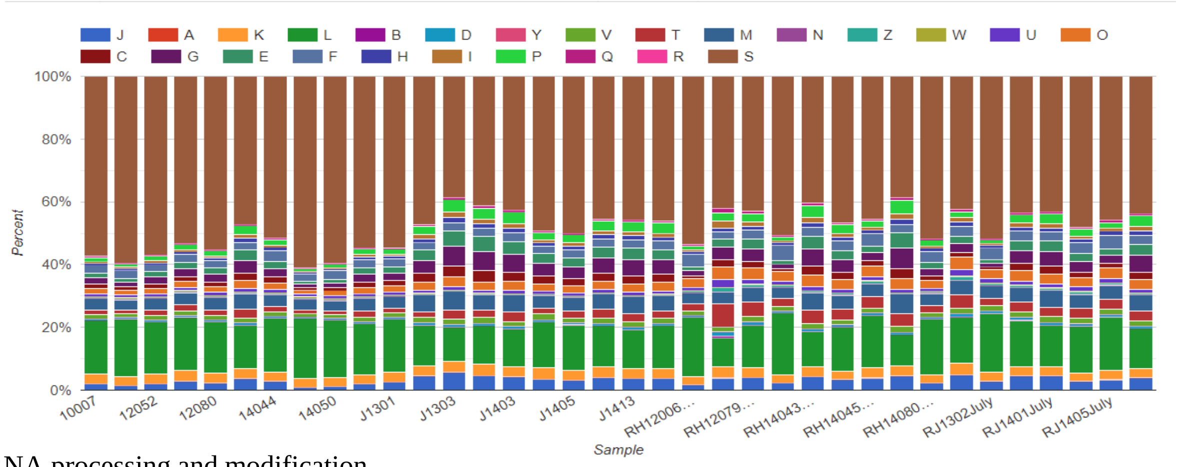

A: RNA processing and modification
B: Chromatin structure and dynamics
C: Energy production and conversion
D: Cell cycle control, Cell division, chromosome partitioning
E: Amino acid transport and metabolism
F: Nucleotide transport and metabolism
G: Carbohydrate transport and metabolism
H: Coenzyme transport and metabolism
I: Lipid transport and metabolism
K: Transcription
J: Translation, ribosome structure and biogenesis
L: Replication, recombination and repair
M: Cell wall/membrane/envelope biogenesis
N: Cell motility
O: Posttranslational modification, protein turnover, chaperones
P: Inorganic ion transport and metabolism
Q: Secondary metabolites biosynthesis, transport and catabolism
R: General function prediction only
S: Function unknown
T: Signal transduction mechanisms
U: Intracellular trafficking, secretion, and vesicular transport
V: Defense mechanism
W: Extracellular structure
Y: Nuclear structure
Z: Cytoskeleton

B

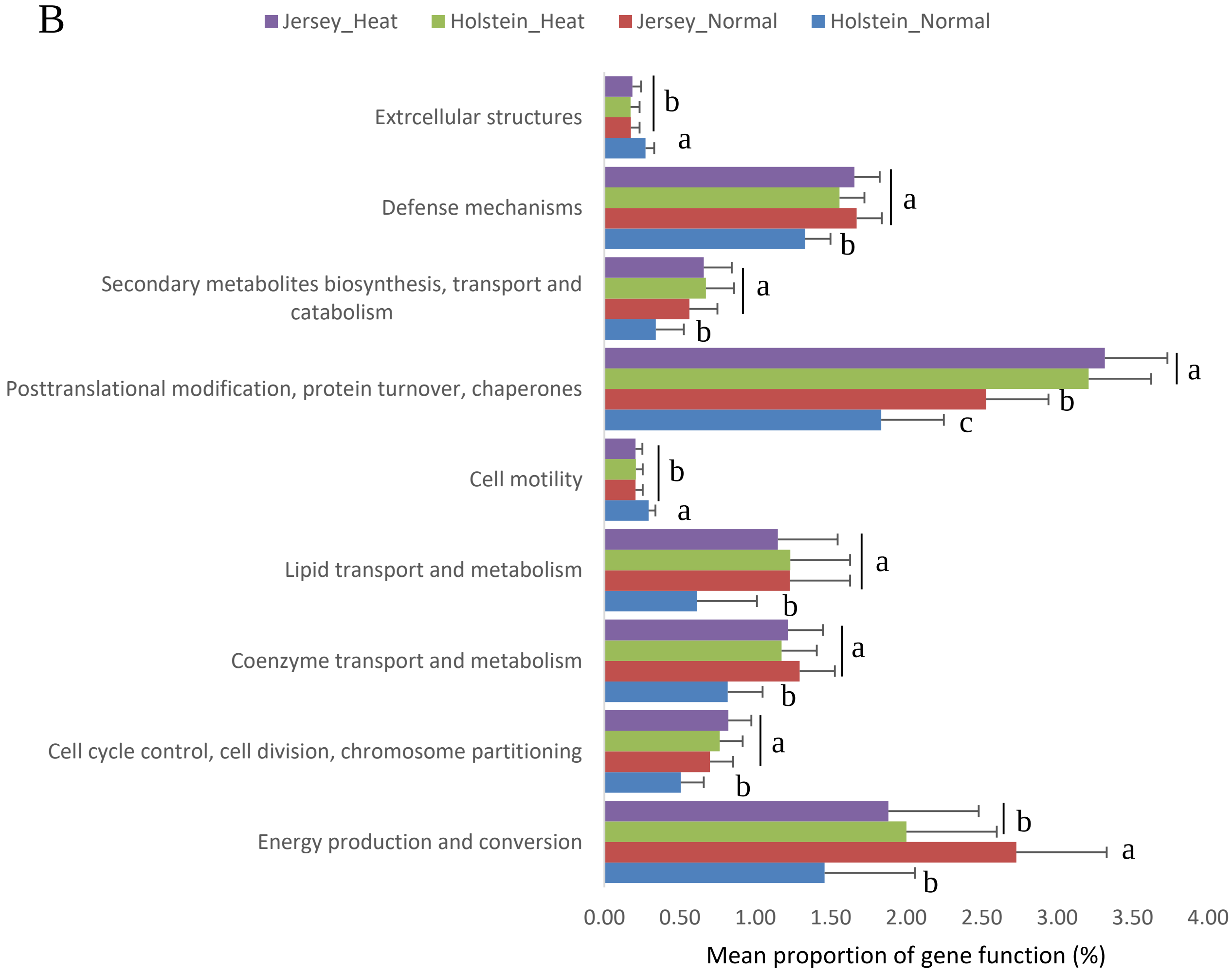

Figure S3. Clusters of Orthologous Groups (COG) distribution of predicted protein domains in rumen samples associated with heat stress obtained from EggNOG database.

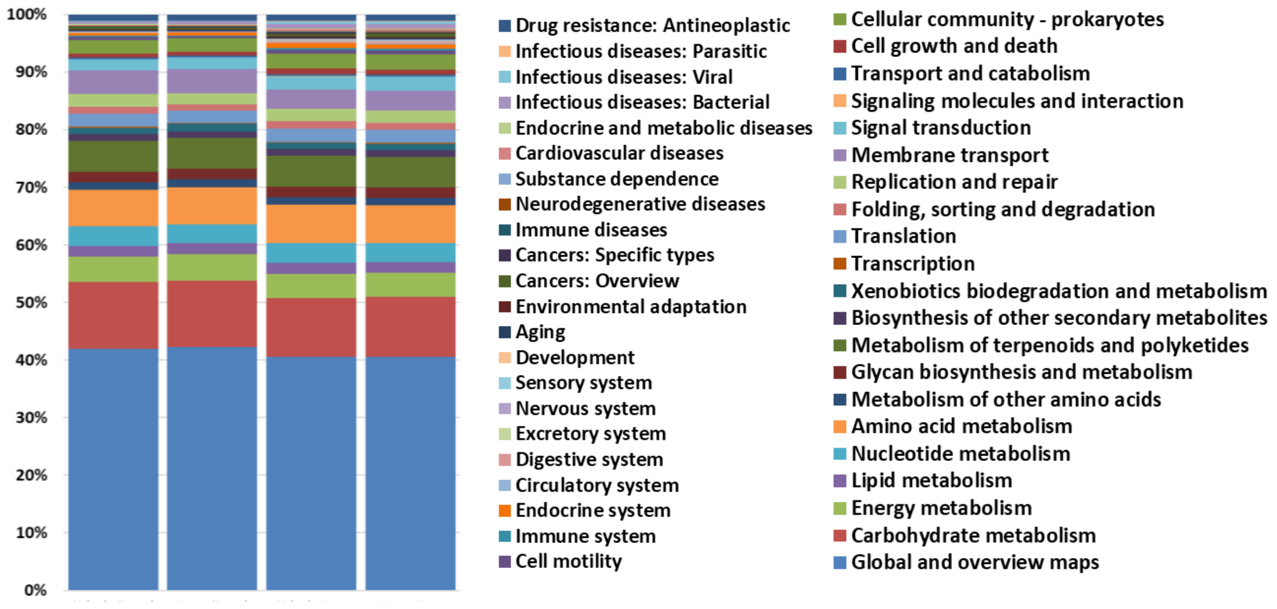

Figure S4. Variations in KEGG metabolic pathways in functional bacterial communities.
